# Etiopathogenesis and Antibacterial Therapy Approach in Patients with Acute Obstructive Pyelonephritis—A Retrospective Study

**DOI:** 10.3390/antibiotics15020164

**Published:** 2026-02-04

**Authors:** Valentin Mitroi, Bogdan Mastalier, Dumitru Dragos Chitca, Andi Fieraru, Iulia Malina Mitroi, Violeta Popovici, Emma Adriana Ozon, Oana Săndulescu

**Affiliations:** 1 Faculty of Medicine, “Carol Davila” University of Medicine and Pharmacy, 050474 Bucharest, Romania; vali_76m@yahoo.com (V.M.); bogdanmastalier@yahoo.com (B.M.); dr.chitca@gmail.com (D.D.C.); oana.sandulescu@umfcd.ro (O.S.); 2 Department of Urology, Colentina Clinical Hospital, 020125 Bucharest, Romania; 3 Department of General Surgery, Colentina Clinical Hospital, 020125 Bucharest, Romania; 4 Department of Urology, Fundeni Clinical Institute, 022328 Bucharest, Romania; fieraruandi@yahoo.com (A.F.); malina.mitroi@gmail.com (I.M.M.); 5 Center for Mountain Economics, “Costin C. Kiritescu” National Institute of Economic Research (INCE-CEMONT), Romanian Academy, 725700 Vatra-Dornei, Romania; 6 Faculty of Pharmacy, “Carol Davila” University of Medicine and Pharmacy, 020945 Bucharest, Romania; emma.budura@umfcd.ro; 7 National Institute for Infectious Diseases “Prof. Dr. Matei Balș”, 021105 Bucharest, Romania; 8 Academy of Romanian Scientists, 050094 Bucharest, Romania

**Keywords:** acute obstructive pyelonephritis, ESBL-producing Enterobacterales, MDR pathogens, antibiotic therapy, urinary drainage, clinical outcomes

## Abstract

**Objectives:** Acute obstructive pyelonephritis (AOP) is a urological emergency that combines bacterial infection with upper urinary tract obstruction. This retrospective study focuses on the microbial etiology and causes of obstruction, clinical manifestations, antibacterial therapy, drainage type, and outcomes in patients diagnosed with AOP at a tertiary urology center between 1 January 2020 and 30 December 2024. **Methods:** One hundred patients with a mean age of 61.30 years were included in this retrospective study, which examines demographic data, comorbidities, clinical features, pathogens involved, antimicrobial regimens, and hospital outcomes. **Results:** Urolithiasis was the most frequent cause of obstruction (62%), followed by ureteral stenosis (14%) and tumors (11%). AOPs were mainly produced by *Escherichia coli* (58%), followed by *Klebsiella* spp. (21%); 18% of all identified bacteria were ESBL-producing Gram-negative bacilli, and 29% were MDR bacteria. The most used IV antibiotics were fluoroquinolones (52%), followed by cephalosporins (19%) and carbapenems (18%). Carbapenems were administered to all patients with AOP caused by ESBL-producing pathogens and to 62% of those with MDR bacteria. The duration of antibiotic therapy was individualized based on clinical response. Switch to oral administration was made after 4.3 ± 1.5 days, and the antibiotic treatment lasted 10.8 ± 3.2 days. **Conclusions:** The results of the present study support integrating evidence-based guidelines with regional patterns of bacterial susceptibility to optimize therapeutic approaches and reduce severe outcomes in patients with AOP, most of whom have multiple comorbidities.

## 1. Introduction

One of the most difficult urological emergencies is acute obstructive pyelonephritis (AOP), which combines mechanical blockage of the collecting system with bacterial infection of the upper urinary tract. The condition is characterized by an acute renal parenchymal infection induced or exacerbated by partial or total obstruction of urine flow, resulting in a synergistic pathological process that is markedly more severe than non-obstructive pyelonephritis. Obstructive pyelonephritis requires immediate surgical or percutaneous intervention in addition to antimicrobial therapy, making it a true urological emergency with mortality rates approaching 70–90% if left untreated [1,2]. This contrasts with simple upper urinary tract infections, which usually respond to antibiotics alone. Urinary stasis and bacterial infection interact to underline the pathophysiology of acute obstructive pyelonephritis. Bacterial growth and retrograde ascent of infection into the renal parenchyma are facilitated when the ureter or renal pelvis is obstructed, whether by calculi, tumors, strictures, or functional failure. When this stasis is coupled with high intrarenal pressure, germs can enter the bloodstream and infiltrate the renal tissue, which frequently leads to acute kidney damage, sepsis, and multi-organ failure. The obstructing element itself may result from a variety of causes, including urolithiasis (which accounts for 62% of cases in the current series), benign or malignant ureteral tumors, ureteral strictures from previous surgery or inflammation, neurogenic bladder dysfunction, or, in rare circumstances, retroperitoneal fibrosis [3,4,5].

### 1.1. Epidemiological Significance and Clinical Burden

In contrast to the female predominance observed in uncomplicated urinary tract infections, the epidemiology of AOP differs significantly from non-obstructive forms. The mean patient age is approximately 62 years, while the sex distribution is relatively balanced (the female percentage is slightly higher than that of males) [6]. The distinct pathophysiological basis of obstructive disease, which is frequently linked to age-related comorbidities like diabetes mellitus, hypertension, and chronic kidney disease, or urological conditions more common in men like benign prostatic hyperplasia, is reflected in this broader age and gender distribution [7]. AOP has clinical importance that extends beyond patient outcomes to encompass broader healthcare and public health issues. The seriousness of this illness is highlighted by rates of significant consequences such as urosepsis (reported in 17% of patients in recent series), acute kidney injury (17.0%), and the need for admission to an intensive care unit (22%) [8]. Even though hospital mortality is low in modern series (roughly 1–2%), the condition’s significant morbidity is concealed by prolonged hospital stays (mean 7.7 days), prolonged antibiotic therapy (mean 10.8 days), and the need for multiple interventions, which significantly increase patient burden and healthcare costs [8]. Additionally, a sophisticated approach to antimicrobial stewardship is required, given the increasing prevalence of multidrug-resistant (MDR) bacteria in urinary tract infections, which affect approximately 29% of patients with acute obstructive pyelonephritis and include extended-spectrum beta-lactamase (ESBL)-producing organisms in 18% of cases [9,10,11,12].

### 1.2. The Challenge of Multidrug-Resistant Bacteria in Acute Obstructive Pyelonephritis

In recent decades, there has been a substantial change in the microbiological landscape of acute obstructive pyelonephritis. ESBL-producing strains and other resistant bacteria are becoming more common, significantly altering treatment algorithms [13], even though *E. coli* remains the most common pathogen, accounting for 58–70% of cases. *P. aeruginosa*, *E. faecalis*, *P. mirabilis*, and *K. pneumoniae* are commonly found, especially in hospitalized individuals or those who have recently been exposed to antibiotics [14,15,16,17,18,19]. The mechanisms of resistance include changes in antibiotic targets, upregulation of efflux pumps, reduced intracellular antibiotic concentrations, modifications to ribosomal targets, and enzymatic degradation of beta-lactam antibiotics via the production of extended-spectrum beta-lactamases and AmpC beta-lactamases [20,21]. These mechanisms of resistance are not merely laboratory findings; they directly translate to clinical failure when inappropriate empirical therapy is selected, prolonged hospitalization, need for escalation to more toxic agents, including carbapenems, and increased risk of adverse outcomes [22,23,24,25,26].

### 1.3. Clinical Emergency and Diagnostic Challenge

The underlying urgency of acute obstructive pyelonephritis is often hidden by its clinical manifestation. Although patients usually exhibit fever (>38.5 °C), unilateral flank pain, and systemic infection symptoms, the severity of the pathological process taking place within the obstructed kidney may not be apparent from the outside. Although fever can be a sign of infection, it may be absent in older or immunocompromised patients, whose clinical presentation may be more subtle, delaying diagnosis and therapy. Clinical judgment, laboratory results, and imaging data must be integrated to differentiate acute obstructive pyelonephritis from uncomplicated acute pyelonephritis or from non-infectious causes of acute flank pain, such as nephrolithiasis without infection or acute myocardial infarction.

Significant leukocytosis (white blood cell count usually >12,000/mm^3^), elevation of acute phase reactants (C-reactive protein > 150 mg/L in most cases), and elevation of procalcitonin (often >2 ng/mL in severe cases) are typical laboratory findings of acute obstructive pyelonephritis [27]. A useful biomarker for the diagnosis and prognosis of acute obstructive pyelonephritis is procalcitonin, a peptide precursor of calcitonin produced mainly by neuroendocrine cells and monocytes in response to bacterial infection and the release of inflammatory cytokines [28]. Procalcitonin levels correlate with the severity of systemic inflammation and the progression of sepsis; concentrations > 2 ng/mL are strongly predictive of bacteremia and sepsis, whereas levels < 0.5 ng/mL or a reduction >80% from baseline indicate resolution of systemic infection [29]. These biomarkers, combined with a urine culture demonstrating significant bacteriuria and urine leukocytosis, establish the infectious component of the diagnosis [30,31,32,33].

The bladder urine culture (PBUC) is the first step in guiding initial antibiotic treatment of patients with AOP. However, urine in the renal pelvis is directly affected by an obstructed and infected urinary system [34]. This often leads to higher bacterial loads and the presence of different, potentially more resistant pathogens than those found in the bladder [35]. To achieve an accurate diagnosis, a renal pelvis urine culture (RPUC) obtained during the surgical decompression is the gold standard [36]. PBUCs are poor predictors of the pathogens present in the obstructed kidney, and there is frequently no correlation between them [35,36].

In AOP, a bladder urine culture typically shows ≥100,000 CFU/mL of a single pathogen [37]. However, lower counts of ≥10,000 CFU/mL may also be clinically significant in the presence of other specific symptoms, still indicating an infection [38,39]. It is important to note that high obstruction can lead to false-negative results in PBUCs. Studies have shown that in severe hydronephrosis, up to 28–50% of patients may have ≤1000 CFU/mL [34,35]. False negatives can occur because complete or severe kidney obstruction may prevent bacteria from reaching the bladder. Up to 31% of patients with AOP have pathogens in the renal pelvis that are completely absent in their bladder urine [40,41]. This can lead to low colony counts or negative PBUCs, even when there are high bacterial loads in the renal pelvis [34].

Renal pelvis urine cultures from patients with AOP, even when colony counts are low, are still considered significant [36,41,42].

To determine the obstructive component of the diagnosis, imaging remains essential. The gold-standard imaging modality for the abdomen and pelvis is computed tomography (CT), with and without intravenous contrast [43]. This modality reveals characteristic findings of hydronephrosis (dilatation of the collecting system), pyonephrosis (dilated collecting system filled with pus), the obstructing lesion itself, and any associated complications, such as renal abscesses or perinephric collections. Acute pyelonephritis may appear on CT as diminished parenchymal enhancement, perinephric fat stranding, and, in more severe cases, gas in the renal parenchyma or collecting system (emphysematous pyelonephritis). The distinction between acute focal bacterial nephritis (a wedge-shaped area of renal hypodensity representing localized suppurative infection without frank liquefaction) and frank renal abscess (a demarcated collection of pus) has significant prognostic implications: the former is typically responsive to antibiotics alone, whereas the latter often requires percutaneous drainage [44].

### 1.4. Historical Context and Evolution of Understanding

Acute obstructive pyelonephritis has been recognized as a distinct clinical entity. Early research highlighted the grave prognosis of untreated obstructed infected kidneys, with observations of rapid progression to systemic toxicity and death [45,46].

The development of imaging modalities—first intravenous pyelography, then ultrasound, and finally CT—revolutionized the diagnosis of obstructive uropathy. It enabled the identification of the blockage combined with infection as a surgical urgency.

A significant advancement was the creation of percutaneous nephrostomy, a minimally invasive method for emergency urine diversion that allowed for the quick decompression of infected occluded kidneys without the morbidity of urgent formal surgical surgery. In certain circumstances, retrograde ureteral stenting has become a viable alternative drainage method in recent times [47,48].

### 1.5. Current Treatment Paradigm: Integration of Drainage and Antimicrobial Therapy

Three fundamental principles underpin the modern management of acute obstructive pyelonephritis: culture-guided antimicrobial therapy, rapid source control via urinary drainage, and supportive care, including fluid resuscitation and hemodynamic optimization. The knowledge that mechanical decompression of the obstructed system must be performed emergently, ideally within 24 to 48 h of presentation, to maximize outcomes arose from the historical recognition that antibiotics alone were insufficient in obstructive pyelonephritis.

Delays in drainage beyond this period are associated with higher mortality, longer sepsis duration, progression to multi-organ failure, and poorer preservation of renal function [46,49].

Rather than being widely accepted, the decision between retrograde ureteral stenting and percutaneous nephrostomy has become more complex and patient-specific. When there is total obstruction, pyonephrosis, severe systemic toxicity, or when anatomical constraints prevent retrograde access, percutaneous nephrostomy has advantages [50].

Direct drainage of infected urine, rapid decompression that reduces kidney injury, direct access for additional intervention (e.g., percutaneous nephrolithotomy for stone removal), and collection of urine specimens for microbiological analysis under direct observation are all made possible by this procedure [51].

Retrograde ureteral stenting, on the other hand, allows patients to return to their usual urine elimination patterns while preserving the internal drainage anatomy and eliminating the need for an external drainage catheter, which carries the risk of infection and dislodgement. Retrograde stenting, however, poses risks of procedure-related bacteremia and ureteral perforation, especially in the context of severe AOP, and may be ineffective in cases of high-grade obstruction [52].

### 1.6. Antimicrobial Stewardship and the Challenge of Empirical Therapy

Given the life-threatening nature of acute obstructive pyelonephritis, the choice of antibacterial therapy must strike a balance between the institutional and societal need to preserve antibiotic efficacy by avoiding needless broad-spectrum use and the necessity of immediate broad-spectrum coverage.

According to current international guidelines, antibiotic therapy includes agents such as piperacillin-tazobactam (which offers broad coverage with beta-lactamase inhibition), carbapenems (especially in settings with a high prevalence of ESBL-producing organisms), or Ceftriaxone (a third-generation cephalosporin that provides excellent renal tissue penetration and activity against most community-acquired Gram-negative pathogens) [53,54,55,56,57,58,59].

### 1.7. The Aim of the Present Study

Recent literature highlights a significant gap in the evidence base regarding the explicit correlation between ESBL-producing pathogen status and key management decisions in acute obstructive pyelonephritis (AOP), including the choice of urinary drainage modality, duration of antimicrobial therapy, and early clinical response. Although ESBL-producing Enterobacterales represent an increasingly prevalent cause of complicated urinary tract infections, with reported prevalence varying widely across geographic regions and healthcare settings [60,61,62], current clinical guidelines and published studies do not provide specific recommendations linking ESBL status to the selection of drainage technique, such as percutaneous nephrostomy versus retrograde ureteral stenting.

In available studies, decisions regarding urgent urinary tract decompression in AOP are primarily guided by the severity of obstruction, presence of pyonephrosis, hemodynamic stability, anatomical considerations, and local expertise [49,63]—factors that are largely independent of the antimicrobial resistance profile of the causative pathogen. Consequently, the potential influence of ESBL production on drainage strategy and early clinical trajectory following decompression remains insufficiently explored.

With respect to antimicrobial therapy, international evidence-based guidelines recommend carbapenems as first-line treatment for ESBL-producing pyelonephritis [64] in settings with high fluoroquinolone resistance or severe infection, while fluoroquinolones or trimethoprim-sulfamethoxazole may be considered when in vitro susceptibility is confirmed [65]. Piperacillin-tazobactam has also been proposed as a potential alternative in selected cases of non-bacteremic ESBL-producing pyelonephritis, based on observational data [66]. However, these recommendations are largely derived from heterogeneous populations and are not specifically tailored to patients with obstructive disease requiring urgent source control.

The optimal duration of antimicrobial therapy in acute obstructive pyelonephritis also remains incompletely defined. Randomized controlled trials in uncomplicated pyelonephritis have demonstrated that shorter courses of fluoroquinolones (5–7 days) are non-inferior to longer regimens [67]. However, evidence guiding treatment duration in complicated and obstructive cases is limited, and therapy is frequently extended to 10–14 days based on clinical judgment rather than high-quality comparative data [68].

Finally, early clinical response markers and biomarker-guided strategies have not been systematically evaluated in obstructive pyelonephritis. Although studies in sepsis and other severe infections have shown that procalcitonin-guided approaches can safely reduce antibiotic exposure without adversely affecting mortality, their applicability to obstructive urinary tract infections remains uncertain. Collectively, these gaps underscore the need for integrated analyses examining how ESBL status relates to drainage choice, antimicrobial duration, and early clinical response in acute obstructive pyelonephritis.

Thus, our retrospective study aims to assess contemporary treatment approaches, patient outcomes, and factors associated with clinical resolution in 100 consecutive patients with acute obstructive pyelonephritis admitted to a tertiary-care urology center between January 2020 and December 2024. The findings highlight that baseline data, comorbidities, obstruction etiology, causative pathogens, and their regional susceptibility profiles strongly influence antibiotic regimens, duration of antibacterial therapy, and short- and intermediate-term outcomes, including complications and intrahospital mortality. This study also integrates biomarkers of systemic inflammation, such as procalcitonin and C-reactive protein, with clinical evaluation and imaging results, aiming to suggest an evidence-based protocol that will direct the intervention type, the choice of antimicrobial agents, and the optimal antibiotic therapy duration—aspects that are still not fully addressed in the scientific literature.

## 2. Results

### 2.1. Sociodemographic and Baseline Data of the Patients with AOP

Patients with AOP (54 women and 46 men) ranged in age from 28 to 87 years, with a mean age of 61.30 (61.44 in females, 61.13 in males; *p* > 0.05; Table 1).

The number of participants with urban residence was 2 times higher than those from rural areas (67% vs. 33%). A negative association was revealed between patients’ gender and residence, although it was not statistically significant (OR < 1, *p* > 0.05, Chi-square; Table 1).

Patients with AOP had different comorbidities: T2DM (38%), CKD (16%), and HTA (47%); more than 20% reported recurrent lithiasis (21%). The incidence of T2DM was higher in women than in men; however, a negative association between T2DM and gender was reported, but it was not significant (OR < 1, *p* > 0.05; Chi-square, Table 1).

Conversely, OR > 1 suggests that hypertension, CKD, and recurrent lithiasis were positively associated with the gender of patients with AOP (*p* > 0.05, Table 1). Positive associations were also reported between T2DM and hypertension (OR = 1.440, *p* = 0.377), T2DM and recurrent lithiasis (OR = 1.293, *p* = 0.606), and between CKD and hypertension (OR = 1.556, *p* = 0.419). None of them were statistically significant (*p* > 0.05).

**Figure 1:**
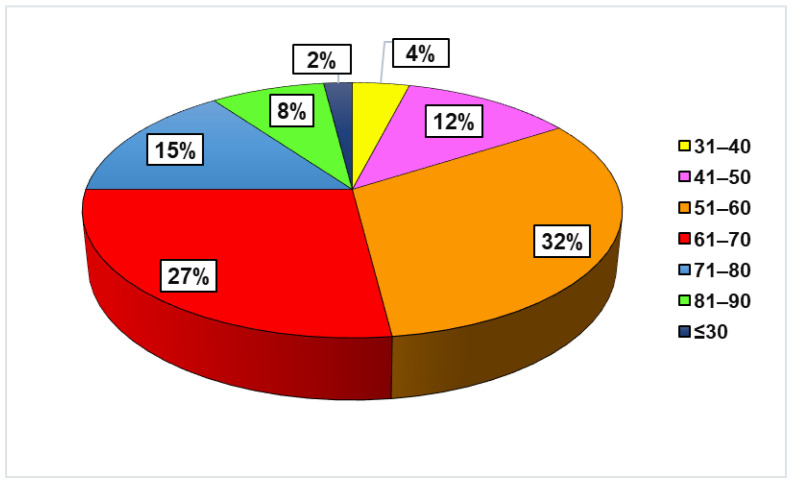
Relative frequencies of age groups (years) in patients with AOP.

Almost 82% of patients with AOP were older than 50 years; the predominant age groups were 51–60 (32%) and 61–70 (27%). Only 2% of participants were aged 30 or younger (Figure 1).

### 2.2. Clinical Manifestations

The essential causes of AOP were lithiasis (62%), ureteral stenosis (14%), tumor (11%), and benign prostate hyperplasia (BPH, 9%), as Table 2 illustrates. Fever (>38.5 °C) was the most common symptom (89%). 71% of patients had lumbar pain, 58% revealed costovertebral tenderness (Giordano sign positive), and 40% claimed dysuria (Table 2). Radiological (Rx) images revealed bilateral involvement (14%), moderate hydronephrosis (grade 2, 42%), and pyonephrosis (32%, Table 2). Among the 32 patients classified as having pyonephrosis in Table 2, imaging evaluation allowed further differentiation of associated parenchymal involvement. Acute focal bacterial nephritis, defined as non-liquefactive parenchymal infection, and frank renal abscess, defined as a liquefied rim-enhancing collection, were recorded as distinct entities and were not included under the definition of pyonephrosis itself.

To better understand the correlations between all these categorical variables, adjusted Pearson residuals (r*ij) and Fisher’s exact test were used.

Thus, a significant negative correlation between ureteral stenosis and HDN 3 (r*ij = −2.329, *p* = 0.019) and a strong association between BPH and pyonephrosis (r*ij = 2.337, *p* = 0.028), were reported. HDN 3 was also associated with hypertension (r*ij = 1.967, *p* = 0.065), while HDN 1 was positively associated with recurrent lithiasis (r*ij = 2.851, *p* = 0.008) and negatively correlated with fever (r*ij = −3.263, *p* = 0.004). In contrast, HDN 4 was significantly associated with lumbar pain (r*ij = 2.010, *p* = 0.05) and pyonephrosis (r*ij = 3.086, *p* = 0.004); lumbar pain was also associated with fever (r*ij = 1.979, *p* = 0.074).

Moreover, dysuria and BPH were strongly associated with males (r*ij = 2.294, *p* = 0.026; r*ij = 3.407, *p* = 0.001, respectively). In contrast, ureteral stenosis was more common in women (r*ij = 2.567, *p* = 0.018).

A significant association was reported between patients aged 51–60 years and the incidence of fever > 38 °C (r*ij = 2.412, *p* = 0.015), 41–50 years and other obstruction causes (r*ij = 2.387, *p* = 0.069), and ureteral stent (r*ij = 2.926, *p* = 0.005), 71–80 years and HDN 2 (r*ij = 2.099, *p* = 0.048), and ≤30 years and pyonephrosis (r*ij = 2.082, *p* = 0.100).

Giordano’s sign (+) was significantly correlated with T2DM (r*ij = 2.905, *p* = 0.006), and bilateral involvement was moderately associated with hypertension (r*ij = 1.975, *p* = 0.081).

### 2.3. AOP Pathogens, Treatment, and Clinical Outcomes

**Figure 2:**
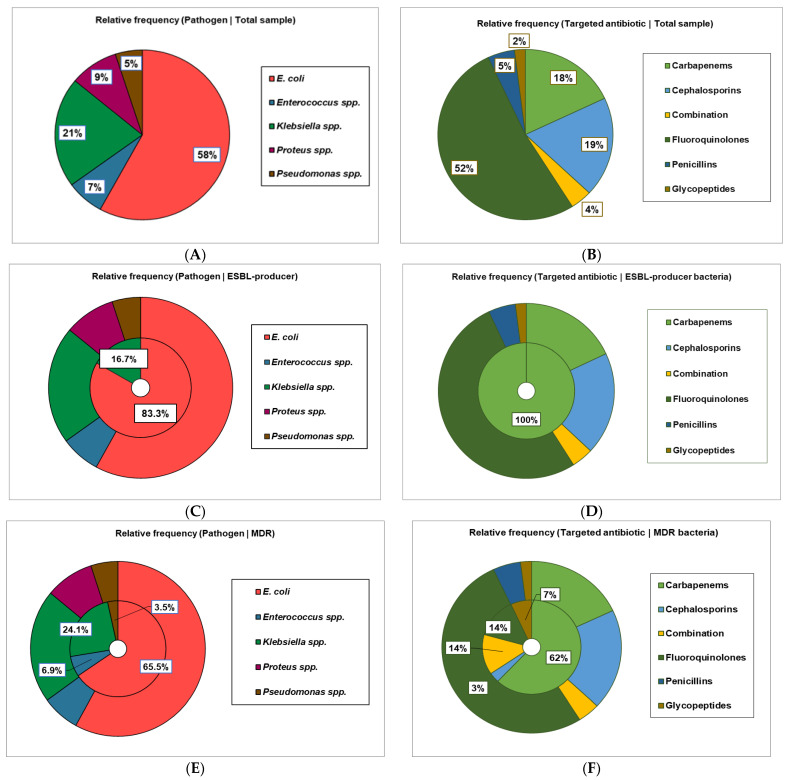
(**A**,**C**,**E**). Bacteria identified in renal pelvis urine cultures of patients with AOP; (**B**,**D**,**F**). Resistance patterns of AOP pathogens: Total group (**A**,**B**); ESBL (+) pathogens (**C**,**D**); MDR bacteria (**E**,**F**).

The most involved pathogen was *E. coli* (58%), followed by *Klebsiella* spp. (21%, Table 3). *Proteus* spp., *Enterococcus* spp., and *Pseudomonas* spp. were each detected in less than 10% patients with AOP (Table 3 and Figure 2A).

Eighteen bacteria from 2 Gram-negative species were identified as ESBL (+) *E. coli* (*n* = 15, 25.86%, r*ij = 2.405, *p* = 0.018, Chi-square test, adjusted Pearson residuals, and Fisher’s exact test), and *Klebsiella* spp. (*n* = 3, 14.29%) (Table 3 and Figure 2C).

Twenty-nine pathogens were MDR; most were *E. coli* (*n* = 19, 32.76%) and *Klebsiella* spp. *(n =* 9, 15.52%). *(n* = 7, 33.33%), followed by *Enterococcus* spp. (*n* = 2, 28.57%) and *Pseudomonas* spp. (*n* = 1, 20%) (Figure 2E). Only *Proteus* spp. is not included in MDR bacteria (r*ij = –2.010, *p* = 0.05; adjusted Pearson residuals and Fisher’s exact test; Figure 2E).

Bacterial species and their ESBL status are essential factors in antibiotic drug selection for the treatment of AOP patients (V = 0.702, *p* < 0.001, V = 0.269, *p* = 0.03; Wilks’ G^2^ test, Table 3).

The most used antibiotics were fluoroquinolones (Ciprofloxacin, 52%), followed by cephalosporins (Cefepime and Ceftriaxone, 19%), carbapenems (Ertapenem and Meropenem, 18%), penicillins (Ampicillin, 5%), combination (Piperacillin-Tazobactam, 4%), and glycopeptides (Vancomycin, 2%) (Figure 2B).

In AOP caused by ESBL (+) pathogens, only carbapenems were administered (r*ij = 10, *p* < 0.0001; adjusted Pearson residuals and Fisher’s exact test; Figure 2D).

However, when MDR bacteria were identified in urine cultures, carbapenems were used in 62% cases (r*ij = 7.33, *p* < 0.0001), followed by combination (represented by piperacillin-tazobactam, 14%, r*ij = 3.19, *p* = 0.006; adjusted Pearson residuals and Fisher’s exact test) and fluoroquinolones (14%), glycopeptides (7%, r*ij = 2.235, *p* = 0.08), and cephalosporins (3%) (Figure 2F).

Adjusted Pearson residuals and Fisher’s exact test strongly supported our results regarding antibacterial therapy, consistent with resistance patterns previously reported in Table 3 and illustrated in Figure 2.

Corresponding statistical data are displayed in Table 4.

Ampicillin, as a sole representative of penicillins, was used only in 71.43% of AOP with *Enterococcus* spp. (r*ij = 8.36, *p* < 0.0001) while in the other 28.57%, the patients received Vancomycin (r*ij = 5.207, *p* = 0.004, Table 4).

From fluoroquinolones, Ciprofloxacin was selected for all AOPs with *Proteus* spp. (r*ij = 3.02, *p* = 0.003) and was preponderantly used in AOPs with *E. coli* (74.14%, r*ij = 5.20, *p* < 0.0001, Table 4).

Over 25% of *E. coli* AOPs were treated with Meropenem, a representative carbapenem (r*ij = 3.57, *p* = 0.000). The other carbapenem, Ertapenem, was administered in 14.29% of AOPs involving *Klebsiella* spp. (r*ij = 3.41, *p* = 0.008, Table 4).

Among cephalosporins, Ceftriaxone was strongly associated with AOPs caused by *Klebsiella* spp. (66.67%, r*ij = 7.82, *p* < 0.0001), while Cefepime was used in all AOPs with *Pseudomonas* spp. (r*ij = 10, *p* < 0.0001, Table 4).

Finally, Piperacillin-tazobactam was included in the treatment of AOPs caused by *Klebsiella* spp. (19.05%, r*ij = 3.95, *p* = 0.002, Table 4).

Drainage methods used were nephrostomy (56%) and ureteral stent (46%). Percutaneous nephrostomy (PCN) was performed in 56 patients with acute obstructive pyelonephritis for emergency urinary tract decompression. The indication for PCN was classified based on radiological and clinical findings. One procedure was performed specifically to drain frank renal abscesses, defined as rim-enhancing cystic parenchymal collections with evidence of liquefactive necrosis that require percutaneous evacuation of purulent material. The remaining 55 procedures were undertaken for emergency decompression of pyonephrosis, defined as infection within a dilated obstructed collecting system without a loculated parenchymal abscess.

Patients diagnosed with acute focal bacterial nephritis (AFBN)—characterized by wedge-shaped or focal non-enhancing parenchymal lesions without liquefaction—accounted for 2 cases among patients with pyonephrosis and were managed conservatively with systemic antimicrobial therapy alone, without the need for separate percutaneous abscess drainage.

Drainage type was significantly associated with bacterial pathogens (OD = 2.640, *p* = 0.005, Fisher’s exact test).

Adjusted Pearson residuals and Fisher’s exact test revealed that *Klebsiella* spp. was associated with nephrostomy (r*ij = 2.097, *p* = 0.048), while *Proteus* spp. was associated with ureteral stent (r*ij = 2.844, *p* = 0.010). Moreover, BPH, as an obstructive cause of AOP, was associated with *Pseudomonas* spp. (r*ij = 2.485, *p* = 0.063).

All ESBL (+) bacteria were MDR (Table 5). Data from Table 5 reveal significant correlations between ESBL and MDR status (V = 1, *p* < 0.0001).

Severe cases requiring ICU admission were reported in 22% patients, and in-hospital mortality was 1% (Table 5).

ESBL status was strongly associated with intrahospital mortality (V = 0.733, *p* = 0.032). It also showed positive correlations with drainage type and ICU admission, but these were not statistically significant (OR > 1; *p* > 0.05; Table 5).

### 2.4. Laboratory Analyses and AOP Evolution

After the Kolmogorov–Smirnov normality test, almost all numerical variables are presented as a mean ± SD in Table 6, grouped by each AOP pathogen. Only 2 variables (PCT and IV switch to oral) were not normally distributed and were reported as median ± Interquartile range (IQR).

*Pseudomonas* spp. was associated with the highest WBC (16,406/mm^3^), while the lowest WBC number/mm^3^ was found in AOP caused by *Enterococcus* spp. (14,211/mm^3^), *p* > 0.05. Patients with AOP caused by *Pseudomonas* spp. had the highest CRP levels (231.10 mg/mL), whereas those with *Proteus* spp. had the lowest (0.00 mg/mL). (182.79 mg/mL, *p* > 0.05, Table 6). PCT values were significantly higher in *E. coli* and *Enterococcus* spp. vs. *Proteus* spp. and *Klebsiella* spp. (5.00 and 6.60 ng/mL vs. 3.90 and 3.70 ng/mL, *p* > 0.05). ESR was highest in AOPs with *Enterococcus* spp. and lowest in those with *Proteus* spp. (68.57 vs. 60.89 mm/h, *p* > 0.05).

Fibrinogen values ranged from 442 mg/dL (*Pseudomonas* spp.) to 515.57 mg/dL (*Enterococcus* spp.), *p* > 0.05 (Table 6). LMR values differed significantly between *E. coli* (3.33) and *Klebsiella* spp. (2.92, *p* > 0.05; Table 6).

The correlations between the categorical variables (from baseline data, clinical manifestations, and imagistic features) and numerical variables (laboratory parameters) were assessed using MANOVA. The statistical results indicate that WBC count (cells/mm^3^) is associated with T2DM (*p* = 0.05), CKD (*p* = 0.08), and lumbar pain (*p* = 0.021). At the same time, CRP levels are correlated with HDN grade (*p* = 0.042), and PCT levels are associated with recurrent lithiasis (*p* = 0.003).

Time to fever resolution (hours) ranged from 55.24 h (*Proteus* spp.) to 64.82 h (*E. coli*; *p* > 0.05). The number of clinical improvement days slightly differs across bacterial species (*p* > 0.05; Table 6). Switching from IV to oral antibiotic therapy (days) decreases from *E. coli* to *Pseudomonas* spp. (*p* > 0.05; Table 6). Hospitalization period (days) and antibiotic treatment duration (days) did not differ significantly (*p* > 0.05; Table 6).

**Figure 3:**
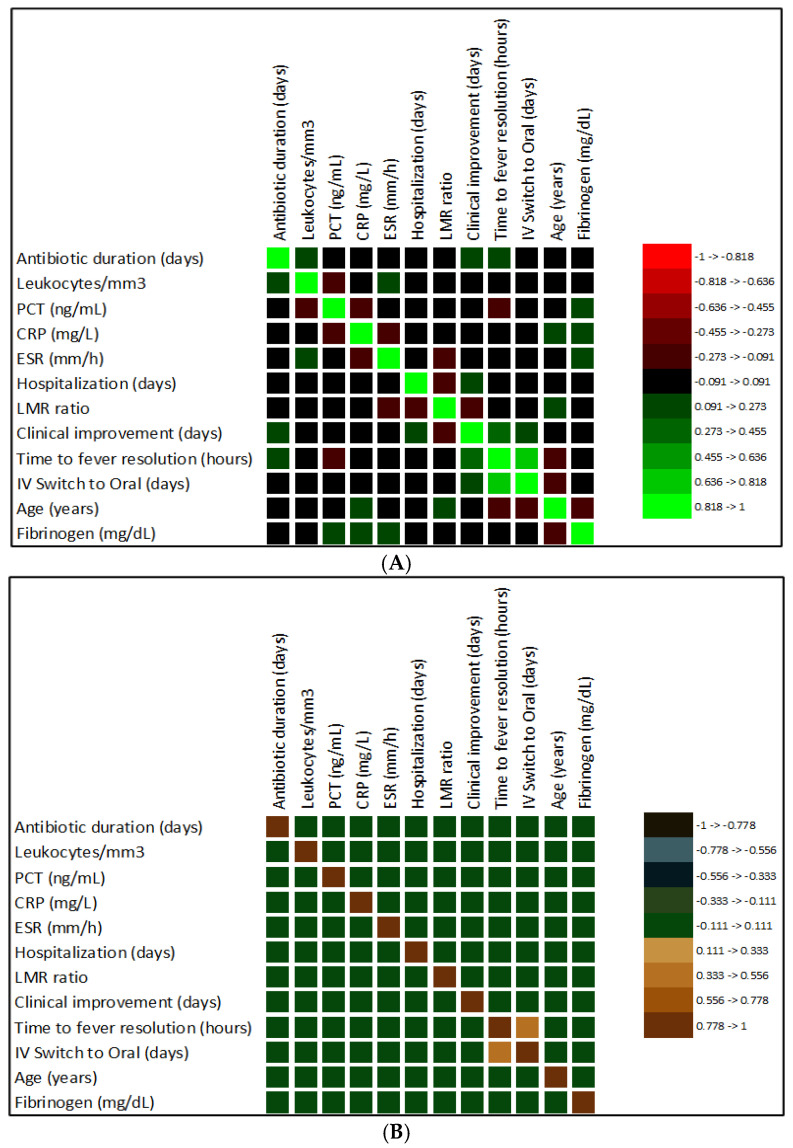
Spearman correlation between laboratory parameters and AOP evolution. (**A**). Image of correlation matrix, based on *r* values (*r* = correlation coefficient); (**B**). Image of the matrix of coefficients of determination (R^2^ = determination coefficient). Different colors correspond to varying ranges of measured coefficient values, as indicated in the legend; CRP—C-reactive protein; PCT—procalcitonin; ESR—erythrocyte sedimentation rate; LMR—lymphocyte-to-monocyte ratio.

To better understand the associations between laboratory parameters and the clinical evolution of patients with AOP, Spearman correlation was performed, and the correlation coefficient (*r*) and determination coefficient (R^2^) were calculated (Figure 3).

Statistical data revealed significant associations between IV-to-oral switch, clinical improvement, and time to fever resolution (*r* = 0.652, *p* < 0.0001; *r* = 0.270, *p* = 0.007; and *r* = 0.317, *p* = 0.001; Figure 3A). The LMR showed a significant negative correlation with clinical improvement (*r* = −0.210, *p* = 0.037; Figure 3A), confirming that a low lymphocyte count is associated with a poor prognosis.

The highest determination coefficients were observed between time to fever resolution and the IV-to-oral switch (R^2^ = 0.450; Figure 3B) and between clinical improvement and the time to fever resolution (R^2^ = 0.100; Figure 3B). These data highlight that clinical improvement and the IV-to-oral switch depend strongly on the time to fever resolution (measured in hours).

The correlations between all categorical variables and numerical parameters related to AOP evolution were examined using MANOVA (Table 7).

Data from Table 7 show that time to fever resolution was strongly associated with the cause of obstruction, fever value, pyonephrosis, and pathogen ESBL and MDR status (*p* < 0.03). In contrast, clinical improvement was correlated with bacterial ESBL status (*p* = 0.003). The switch from IV to oral antibiotic therapy was influenced by ESBL and MDR status of pathogens, hydronephrosis, bilateral involvement, recurrent lithiasis, and residence (*p* < 0.05). The number of days of antibacterial therapy was associated with the cause of obstruction and residence (*p* < 0.04). Moreover, ESBL status was a key factor influencing the early stages of AOP evolution and the transition from IV to oral antibiotic therapy (*p* ≤ 0.003).

## 3. Discussion

### 3.1. Summary of Principal Findings

This single-center retrospective cohort of 100 consecutive patients with acute obstructive pyelonephritis provides contemporary, granular data on microbiological profiles, antibacterial resistance patterns, antibiotic treatment strategies, and clinical outcomes in a tertiary-care setting.

*Escherichia coli* was the predominant uropathogen (58%), and the prevalence of ESBL-producing Enterobacterales (18% overall) and multidrug-resistant organisms (29%) was substantial, reflecting the global proliferation of antimicrobial resistance in complicated urinary tract infections. Patients with MDR infections had a significantly higher rate of carbapenem use (65.5% of MDR patients vs. 7.0% of non-MDR patients, *p* < 0.001), indicating that broad-spectrum agents should be appropriately targeted to resistant pathogens while minimizing exposure in susceptible infections—a crucial antimicrobial stewardship principle. Carbapenem therapy was administered to over 20% of patients at some point during their treatment. Over 50% of patients received fluoroquinolones (IV). Ciprofloxacin was also administered orally for step-down therapy when intravenous agents lacked oral bioequivalent agents (e.g., Ceftriaxone, Meropenem, Ertapenem, Piperacillin–Tazobactam, Vancomycin). It allowed clinically stable patients with sensitive isolates to switch from intravenous to oral dosing early (median 3.8 days, mean 4.3 ± 1.5 days). The mean antibiotic duration was 10.8 ± 3.2 days and did not significantly differ by ESBL status (ESBL-positive: 10.7 days vs. ESBL-negative: 10.9 days). It suggests that treatment duration was appropriately tailored to clinical response rather than empirically extended for resistant organisms or severe presentations.

### 3.2. Microbiological Landscape: Extended-Spectrum β-Lactamases and Multidrug Resistance in Acute Obstructive Pyelonephritis

The 18% ESBL prevalence and 29% MDR prevalence in our cohort reflect the current state of antimicrobial resistance in complicated urinary tract infections presenting to tertiary care centers in Romania. These figures align closely with regional surveillance data from Eastern Europe and mirror global trends toward escalating resistance. According to the European Centre for Disease Prevention and Control (ECDC), Romania ranks among the countries with the highest resistance rates in Europe, with carbapenem resistance in *K. pneumoniae* increasing from 7.12 per 100,000 population in 2019 to 20.02 per 100,000 in 2023 (181.2% increase) [69,70].

The predominance of *E. coli* among ESBL producers is consistent with molecular epidemiology demonstrating that CTX-M-type ESBLs—the most prevalent ESBL family globally—are particularly common in *E. coli*. The ESBL rate among *E. coli* in our series exceeds the approximately 15–20% rates reported in Western Europe and North America but aligns with the 25–45% rates documented in Eastern Europe, the Mediterranean region, and much of Asia. A recent Romanian study of dermatology patients reported ESBL prevalence of 46.1% for *E. coli* and 66.0% for *Klebsiella* spp. across various clinical specimens, suggesting even higher resistance rates in specific patient populations. This geographic variation reflects differences in antibiotic consumption patterns, infection control practices, and healthcare infrastructure [70,71,72].

### 3.3. Carbapenem Use and Stewardship: The Central Tension

The 18% overall carbapenem exposure in our cohort is substantial and merits critical examination from a stewardship perspective. Carbapenems are classified as “Watch” antibiotics by the World Health Organization AWaRe framework, indicating they should be prioritized as targets for stewardship programs due to their high resistance potential, and as “last-resort” agents whose overuse drives carbapenem-resistant Enterobacterales, which are designated as “critical priority” pathogens for which few treatment options remain [69,70,73]. Moreover, antimicrobial resistance among non-fermenting Gram-negative bacilli (including *Pseudomonas aeruginosa*) is a major cause of opportunistic septicemia in intensive care units (ICUs), primarily affecting immunocompromised or critically ill patients who undergo invasive procedures [74,75,76].

Nonetheless, opportunities for further carbapenem-sparing exist. Piperacillin–tazobactam, used in only 4 patients in our series, represents a potential carbapenem alternative for non-bacteremic ESBL urinary tract infections when MICs are low (≤8–16 mg/L) and high-dose, extended-infusion regimens are utilized. Multiple retrospective cohort studies and meta-analyses have demonstrated non-inferiority of piperacillin–tazobactam compared to carbapenems specifically for ESBL urinary sources (pyelonephritis, cystitis), with microbiological cure rates of 85–95% and clinical cure rates of 90–95%, provided appropriate dosing (4 g + 0.5 g every 6 h as a 4 h infusion) is employed [77].

### 3.4. Duration of Therapy: Shorter Courses and Biomarker Guidance

The 10.8 ± 3.2-day mean antibiotic duration in our cohort falls within guideline-recommended ranges for complicated UTI (7–14 days) but is shorter than historical practices, which often employed 14–21 days for pyelonephritis regardless of clinical response. Our clinicians individualized antibiotic therapy period based on clinical response—an approach consistent with emerging evidence supporting shorter, response-guided courses [78].

Multiple randomized controlled trials have demonstrated that 7–10-day courses achieve outcomes equivalent to 14-day courses for complicated UTI when source control is adequate. A landmark study randomized 504 patients with uncomplicated Gram-negative bacteremia (predominantly urinary source) to CRP-guided duration versus fixed 7 days versus fixed 14 days, demonstrating non-inferiority of both CRP-guided (median 7 days) and fixed 7-day therapy compared to 14 days, with 30-day failure rates of 2.4%, 6.6%, and 5.5%, respectively [79,80]. Although this trial enrolled primarily non-obstructive bacteremia, the principle that shorter durations are safe when clinical response is documented is increasingly accepted for obstructive infections once drainage is achieved [81,82,83]. Moreover, biomarker-guided duration using PCT and/or CRP represents an evidence-based strategy to individualize therapy while minimizing overtreatment [78,84,85].

The mean duration of antimicrobial therapy in this cohort (10.8 ± 3.2 days) reflects contemporary clinical practice for acute obstructive pyelonephritis, which is classified as a complicated urinary tract infection because of upper urinary tract obstruction. Although randomized trials have demonstrated that shorter courses of fluoroquinolones (i.e., 7 days) are noninferior to longer regimens in uncomplicated acute pyelonephritis, these findings cannot be directly extrapolated to obstructive disease, where impaired urinary drainage and delayed source control contribute to increased bacterial burden and infection severity [65].

Several characteristics of the present cohort support the observed treatment duration. First, all patients had anatomically defined obstruction, most commonly due to urolithiasis, ureteral stenosis, or malignant compression, conditions known to impair bacterial clearance and necessitate urgent decompression. Second, a substantial proportion of patients had comorbidities associated with delayed resolution of infection, including diabetes mellitus, chronic kidney disease, and cardiovascular disease. Third, resistant pathogens were frequently identified, with ESBL-producing Enterobacterales and multidrug-resistant organisms accounting for a significant proportion of isolates, often necessitating the use of broad-spectrum agents typically used for longer treatment courses in complicated infections [86].

In addition, a high proportion of patients presented with pyonephrosis or parenchymal involvement, including acute focal bacterial nephritis and suspected renal abscess formation, entities that represent more severe infectious phenotypes than uncomplicated pyelonephritis. Taken together, these factors—obstructive anatomy, comorbid conditions, resistant organisms, and increased infection severity—justify the mean antibiotic duration observed in this study and are consistent with guideline-supported, individualized treatment strategies for complicated urinary tract infections following adequate source control.

### 3.5. Limitations

Several limitations of this study should be mentioned, as follows:

○ The retrospective, single-center design limits generalizability and introduces potential selection bias.
○ Patients presenting to a tertiary referral center in Romania may have more severe illness, higher comorbidity burden, and greater resistance prevalence than community hospital cohorts, potentially inflating our ESBL and MDR rates. Prospective, multicenter validation is needed to confirm our findings in diverse practice settings.
○ This retrospective study includes only the AOP patients with bacteriuria > 100.000 CFU/mL in renal pelvis urine cultures.
○ The absence of molecular characterization of ESBL types (CTX-M, TEM, SHV variants) and other resistance mechanisms precludes detailed epidemiological analysis of resistance determinants. Knowledge of circulating ESBL subtypes and plasmid types would inform infection control interventions and enable comparison to regional surveillance data [87].
○ The study did not systematically capture antibiotic adverse events beyond major complications requiring treatment discontinuation, thereby limiting the assessment of the safety-efficacy trade-off across different regimens. Fluoroquinolone-associated tendon, neurological, and cardiovascular toxicities; carbapenem-associated seizures and *Clostridioides difficile* infection; and other antibiotic-related harms are essential considerations in treatment selection but were not comprehensively recorded.
○ A cost-effectiveness analysis comparing different antibiotic strategies (e.g., empirical Ceftriaxone vs. carbapenems; IV-only vs. early oral step-down) was not performed, despite healthcare costs being a major driver of stewardship initiatives.

### 3.6. Future Research Directions

As a research perspective, several priorities emerge.

Randomized controlled trials comparing piperacillin–tazobactam to carbapenems specifically for non-bacteremia ESBL pyelonephritis would provide definitive evidence for carbapenem-sparing strategies.

Prospective validation of biomarker-guided duration algorithms in AOP using PCT and/or CRP discontinuation thresholds could establish standardized stewardship approaches [85]. Recent studies evaluated IV Fosfomycin (alone or in combination) [88,89], Cefepime-Enmetazobactam [90], Ceftazidime-Avibactam [91], and oral Minocycline (as a new option for step-down therapy for ESBL upper urinary tract infections) [92]. Therefore, therapeutic options for resistant pathogens implicated in AOP etiology could be expanded.

Emerging evidence suggests that inflammatory biomarkers may facilitate safe reductions in antibiotic exposure in selected infectious syndromes [93]. In sepsis and other severe bacterial infections, procalcitonin-guided antibiotic discontinuation strategies have been shown to shorten treatment duration by approximately 1–2 days without adversely affecting mortality or clinical outcomes [94,95]. Large, randomized trials and meta-analyses have demonstrated consistent reductions in antibiotic exposure using biomarker-guided approaches compared with standard care [96].

However, data specifically addressing the duration of antibiotic therapy guided by biomarkers in acute obstructive pyelonephritis remain limited. Given that all patients in the present cohort had measurements of procalcitonin and C-reactive protein, biomarker-guided strategies offer a potential avenue for future prospective studies to optimize antibiotic duration after effective urinary tract decompression while minimizing unnecessary antimicrobial exposure and the risk of resistance development.

Investigating the barriers to implementing stewardship interventions in urological emergencies (de-escalation protocols, oral step-down pathways, biomarker algorithms) would facilitate the broader dissemination of evidence-based practices.

Finally, health economic analyses comparing costs, quality-adjusted life-years, and the effects of antimicrobial resistance across different AOP treatment strategies would inform resource allocation and policy decisions.

## 4. Materials and Methods

### 4.1. Study Design and Patient Population

One hundred consecutive patients with AOP who received treatment at the Department of Urology, Colentina Clinical Hospital, Bucharest, Romania, between January 2020 and December 2024 were included in this retrospective observational study. Clinical outcomes, microbiological profiles, antibiotic efficacy, and variables associated with successful clinical resolution were assessed in the study. The Declaration of Helsinki’s ethical guidelines were followed in all study methods (Fortaleza revision, 2013). Before data collection, institutional review board consent was obtained, and patient anonymity was ensured by assigning numeric codes that did not retain personally identifiable information.

The research was conducted in accordance with the principles of the Declaration of Helsinki (Fortaleza revision, 2013). Institutional ethics committee approval was obtained before initiation (Protocol Approval Reference: 22, dated 23 July 2025). For the retrospective cohort component, patient data were anonymized entirely through numerical coding, without retention of personally identifiable information, and a waiver of informed consent was required per institutional policy for retrospective minimal-risk research. All patient data were stored on a secure, password-protected institutional server with access restricted to authorized research team members. Data management fully complied with the General Data Protection Regulation (EU 2016/679--GDPR), with patient information maintained in confidence throughout the study and archived in accordance with institutional protocol for a minimum of 5 years after study completion.

#### 4.1.1. Inclusion/Exclusion Criteria

Inclusion criteria were as follows:

(1) Diagnosis of acute obstructive pyelonephritis confirmed by clinical presentation (fever ≥ 38.5 °C, flank pain, costovertebral angle tenderness), laboratory findings (elevated white blood cell count > 12,000/mm^3^, elevated C-reactive protein CRP > 50 mg/L, positive renal pelvis urine culture with >100,000 colony-forming units per milliliter (CFU/mL)), and imaging (computed tomography or ultrasound confirming hydronephrosis and ureteral/pyelocaliceal obstruction);
(2) Age ≥ 18 years at presentation in the emergency care unit;
(3) Receipt of urinary drainage intervention (percutaneous nephrostomy or retrograde ureteral stenting);
(4) Administration of systemic antimicrobial therapy according to institutional protocols.

Exclusion criteria consisted of:

(1) Chronic kidney disease stage 5 (estimated glomerular filtration rate [eGFR] < 15 mL/min/1.73 m^2^);
(2) Renal transplantation history;
(3) Uncomplicated urinary tract infections without documented obstruction;
(4) Incomplete clinical, laboratory, or imaging data;
(5) Refusal of standard therapeutic procedures.

Of 134 patients screened during the study period, 100 met the inclusion criteria and were enrolled in the analysis.

#### 4.1.2. Data Collection

Clinical data were systematically extracted from institutional medical records and standardized case report forms.

Documented demographic information included age, biological sex, and residence.

Comorbid conditions assessed included type 2 diabetes mellitus, arterial hypertension, chronic kidney disease (defined as eGFR < 60 mL/min/1.73 m^2^ on two or more occasions ≥ 90 days apart), and recurrent urolithiasis (defined as ≥2 episodes of stone formation in the preceding 5 years).

Clinical presentation features documented were: (1) fever > 38.5 °C; (2) lumbar pain (flank pain); (3) Giordano sign positivity (costovertebral angle tenderness elicited by percussion); and (4) dysuria or lower urinary tract symptoms. Obstruction etiologies were categorized as: urolithiasis, ureteral strictures, ureteral tumors, benign prostatic hyperplasia with retention, neurogenic bladder dysfunction, or other anatomical abnormalities.

### 4.2. Drainage Methods

The urologist decided between retrograde ureteral stenting and percutaneous nephrostomy based on the patient’s clinical status, institutional expertise, anatomical considerations, and the degree of obstruction, as part of routine clinical practice [97].

### 4.3. Clinical Laboratory Investigations

#### 4.3.1. Hematologic and Biochemical Parameters

These parameters were measured at hospital admission. They included complete blood cell count (white blood cell count in cells/mm^3^, absolute lymphocyte count, absolute monocyte count, hemoglobin).

Lymphocyte-to-monocyte ratio (LMR) was calculated as absolute lymphocyte count divided by absolute monocyte count.

#### 4.3.2. Biomarkers of Systemic Inflammation

○ C-reactive protein (CRP), measured by high-sensitivity turbidimetric immunoassay, with results expressed in mg/L;
○ Procalcitonin (PCT), measured by immunoluminometric assay with results expressed in ng/mL, with a lower limit of detection of 0.02 ng/mL;
○ Erythrocyte sedimentation rate (ESR), measured by the Westergren method in mm/h;
○ Fibrinogen, measured by the Clauss method in mg/dL.

#### 4.3.3. Urine Analysis

Urine analysis was performed on all patients and documented the presence of pyuria (≥5 white blood cells/high-power field), bacteriuria (≥1+ on microscopy), hematuria (red blood cells present), and nitrites or leukocyte esterase positivity.

Two urine specimens were collected. Preoperatively, a bladder urine sample was obtained via midstream clean catch or straight catheterization before antibiotic administration, using standard techniques to minimize contamination. The renal pelvis urine specimen was collected during the surgical decompression. Urine cultures (PBUC and RPUC) were performed on blood agar and MacConkey agar plates, and identification and antimicrobial susceptibility testing were performed using automated systems.

Only patients with bacterial colony counts > 100,000 CFU/mL in RPUCs were included in our retrospective study.

Antimicrobial susceptibility testing was performed using the EUCAST disk diffusion method, in accordance with the European Committee on Antimicrobial Susceptibility Testing guidelines (EUCAST, version 13.0, 2023, http://www.eucast.org). Interpretation of inhibition zone diameters and minimum inhibitory concentrations was based on EUCAST clinical breakpoint tables. Antibiotic susceptibility testing was performed using the following agents: Ampicillin, Amoxicillin + Clavulanic acid, Piperacillin-Tazobactam, cephalosporins (Ceftriaxone, Ceftazidime, Cefepime), fluoroquinolones (Ciprofloxacin, Levofloxacin), carbapenems (Imipenem, Meropenem, Ertapenem), aminoglycosides (Gentamicin, Amikacin), and Vancomycin.

Extended-spectrum beta-lactamase (ESBL) production among Enterobacterales was detected using the double-disk synergy test [98], with third-generation cephalosporin disks (cefotaxime and/or ceftazidime).

Phenotypic confirmation of ESBL production was performed using the combined disk method with clavulanate, in accordance with EUCAST recommendations. An increase in the inhibition zone diameter of ≥5 mm for cephalosporin disks in the presence of clavulanate, compared with disks without inhibitor, was interpreted as confirmatory for ESBL production.

MDR was defined as acquired non-susceptibility to at least one agent in three or more antibiotic drug categories [99].

### 4.4. Imagistic Investigations

#### 4.4.1. Renal Ultrasound

Renal ultrasound was performed within 2 h from presentation using high-resolution B-mode ultrasound (Alpinion E-Cube i7, Alpinion Medical Systems, Seoul, Republic of Korea) to assess: presence and degree of hydronephrosis (graded as Grade I—mild pelvic dilatation, Grade II—moderate with calyces blunted, Grade III—severe with marked caliceal dilatation, or Grade IV—massive with parenchymal thinning); renal size and echotexture; presence of focal or diffuse alterations in parenchymal echogenicity and perinephric fluid collection.

#### 4.4.2. Computed Tomography (CT)

CT of the abdomen and pelvis with intravenous iodinated contrast (0.03 mL/kg at 300–320 mg I/mL) was achieved as the definitive imaging modality for all patients. Unenhanced (pre-contrast) images were obtained from the dome of the diaphragm to the pubic symphysis, followed by acquisition of images at 25–30 s (arterial phase) and 60–70 s (delayed/excretory phase) after bolus injection of contrast material. CT findings were assessed for:

○ Obstruction location and etiology, including stone size (largest dimension in millimeters), stone density in Hounsfield units (HU), ureteral stricture caliber, mass characteristics;
○ Hydronephrosis grade (as defined above);
○ Renal parenchymal enhancement pattern (homogeneous versus heterogeneous, measured as the difference in HU between arterial and delayed phase);
○ Perinephric fat stranding (defined as abnormal attenuation of perinephric fat);
○ Presence of pyonephrosis, defined as gas-fluid levels, thick purulent collection, or clinical correlation with fever and elevated inflammatory markers;
○ Renal abscess, defined as a focal fluid collection (15–30 HU) with peripheral enhancement and rim restriction on diffusion-weighted imaging (if MRI obtained);
○ Presence of emphysematous pyelonephritis, defined as gas within renal parenchyma or collecting system. According to Mackler’s classification, 4 classes were identified: Class 1—gas in the collecting system; Class 2—gas in the renal parenchyma; Class 3—extension into the perinephric space; Class 4—gas in the retroperitoneal space; and Class 5—gas in the renal and perirenal spaces.

#### 4.4.3. Magnetic Resonance Imaging (MRI)

It is performed when CT findings are equivocal or to further characterize complex anatomy, using T2-weighted imaging for anatomical detail and diffusion-weighted imaging (DWI) with apparent diffusion coefficient (ADC) mapping to identify areas of restricted diffusion suggestive of abscess or severe infection.

### 4.5. Antibacterial Therapy

Antimicrobials used in routine clinical practice for IV administration in the urology department included Ciprofloxacin (400 mg every 12 h), Ertapenem (1 g every 24 h), Meropenem (1 g every 8 h), Ceftriaxone (1 g every 12 h), Cefepime (1–2 g every 12 h), Piperacillin-Tazobactam (4 g/0.5 g every 8 h), Ampicillin (2 g every 4–6 h), and Vancomycin (1 g every 12 h).

Ampicillin was selected against susceptible *Enterococcus* spp. At the same time, Vancomycin was administered for suspected Ampicillin-resistant *Enterococcus* spp., although this organism was rarely encountered in this series. Ertapenem was reserved for AOP in cases of pathogens susceptible to carbapenems but resistant to cephalosporins.

Oral step-down therapy was initiated when the patient’s status was deemed appropriate, defined as clinical improvement, normalization of vital signs, and reduced inflammatory markers. A proper oral antimicrobial drug confirmed to inhibit the identified pathogens was regularly selected, as follows:

○ Cephalosporins (Cefixime 200–400 mg every 12 h, Cefuroxime 500 mg every 12 h, Cefaclor 500 mg every 8 h, and Cephalexin 500 mg every 6 h);
○ Fluoroquinolones (Ciprofloxacin 500 mg every 12 h, Levofloxacin 500 mg every 24 h, and Norfloxacin 400 mg every 12 h);
○ Penicillins (Amoxicillin 1 g every 8 h)

Duration of antimicrobial therapy was determined in routine clinical practice by: (1) clinical response (resolution of fever, improvement in hemodynamic parameters); (2) laboratory markers (CRP reduction ≈ 75% from peak or achievement of <20 mg/L [85]; PCT reduction > 80% from peak or achievement of <0.5 ng/mL); (3) imaging criteria (normalization of hydronephrosis grade on ultrasound, resolution of perinephric stranding on CT).

### 4.6. Statistical Analysis

All analyses were performed using XLSTAT Premium 2025 v. 2.0.1432 by Lumivero (Denver, CO, USA) [100]. Initially, the distribution of all quantitative data was analyzed using the Kolmogorov–Smirnov test (usually recommended for clinical studies with over 50 participants).

Then, descriptive statistics were used to summarize the characteristics of the patients with AOP included in this study. Continuous variables with normal distribution were expressed as mean ± SD (standard deviation), and the statistically significant differences were investigated using a single-factor ANOVA. Those that did not follow normal distribution were expressed as medians with interquartile ranges (IQRs).

Categorical variables were reported as counts (*n*) and relative frequencies (%). Chi-square test and Wilks’ G^2^ test (with Odds Ratio (OD) and Cramer’s V (V) as association coefficients). Adjusted Pearson residuals (r*ij), and Fisher’s exact test were also applied to categorical variables [101], to assess independence across different data groups. Values of r*ij > ±1.96 indicate a statistically significant association [102].

The Spearman correlation was used to assess the relationship (correlation and determination) between quantitative variables (laboratory parameters and AOP evolution).

The predictive correlations between categorical variables and quantitative variables were evaluated using MANOVA [103].

For all analytical tools, statistical significance was assessed at *p* ≤ 0.05.

## 5. Conclusions

The present study highlights the complex management of patients with AOP, underscoring the importance of a multidisciplinary approach that integrates early diagnostic tools with well-established rapid drainage and antibiotic therapy based on regional antibacterial susceptibility patterns. By combining these perspectives, further research could develop more sensitive and specific diagnostic markers for early detection and targeted antibacterial treatment.

## Figures and Tables

**Table 1:** Sociodemographic and baseline data of the patients with AOP.

Data		Total		F		M		*p*-Value	OR
		100		54		46			
Data Report/ Statistical tool	Age	Mean	SD	Mean	SD	Mean	SD	ANOVA	
		61.3	12.26	61.44	13.28	61.13	10.98	>0.05	-
		*n*	%	*n*	%	*n*	%	Chi-square test	
Residence	Rural	33		16	29.63	17	36.96	0.437	0.718
	Urban	67		38	70.37	29	63.04		
T2DM	0	62		32	59.26	30	65.22	0.541	0.776
	1	38		22	40.74	16	34.78		
Hypertension	0	53		29	53.70	24	52.17	0.879	1.063
	1	47		25	46.30	22	47.83		
CKD	0	84		48	88.89	36	78.26	0.148	2.222
	1	16		6	11.11	10	21.74		
RL	0	79		43	79.63	36	78.26	0.867	1.086
	1	21		11	20.37	10	21.74		

*n*—frequency, %—relative frequency; SD—standard deviation; F—female; M—male; *p* < 0.05—statistically significant differences; T2DM—Type 2 Diabetes mellitus; CKD—chronic kidney disease; RL—recurrent lithiasis; 0—No, 1—Yes; OR—odd ratio, a statistical measure used to quantify the strength and direction of the association between two categorical variables: positive correlation (OD > 1), negative correlation (OD < 1), no correlation (OD = 1), according to https://rpubs.com/snijesh/odds-ratio, accessed on 28 January 2026.

**Table 2:** Clinical manifestations at ECU admission and radiological features.

Parameter	Total	BPH		Lithiasis		Tumor		Ureteral Stenosis		Other	
	*N* = %	*n*	%	*n*	%	*n*	%	*n*	%	*n*	%
	100	9		62		11		14		4	
**Symptoms**											
Fever (>38.5 °C)											
0	11	1	11.11	8	12.90	0	0.00	2	14.29	0	0.00
1	89	8	88.89	54	87.10	11	100.00	12	85.71	4	100.00
Lumbar pain											
0	29	1	11.11	19	30.65	3	27.27	5	35.71	1	25.00
1	71	8	88.89	43	69.35	8	72.73	9	64.29	3	75.00
Giordano sign											
GS (+)	58	6	66.67	35	56.45	6	54.55	8	57.14	3	75.00
GS (−)	42	3	33.33	27	43.55	5	45.45	6	42.86	1	25.00
Dysuria											
0	60	5	55.56	38	61.29	4	36.36	10	71.43	3	75.00
1	40	4	44.44	24	38.71	7	63.64	4	28.57	1	25.00
**Imagistic features**											
Bilateral involvement											
0	86	7	77.78	54	87.10	8	72.73	13	92.86	4	100.00
1	14	2	22.22	8	12.90	3	27.27	1	7.14	0	0.00
Hydronephrosis grade (1–4)											
HDN 1	24	1	11.11	14	22.58	3	27.27	5	35.71	1	25.00
HDN 2	42	5	55.56	23	37.10	5	45.45	7	50.00	2	50.00
HDN 3	25	2	22.22	19	30.65	3	27.27	0	0.00	1	25.00
HDN 4	9	1	11.11	6	9.68	0	0.00	2	14.29	0	0.00
Pyonephrosis											
0	68	3	33.33	46	74.19	7	63.64	9	64.29	3	75.00
1	32	6	66.67	16	25.81	4	36.36	5	35.71	1	25.00

ECU—Emergency Care Unit, GS—Giordano sign (Costovertebral tenderness); HDN—Hydronephrosis; all features are categorical variables and were recorded as a patient’s number (*n*) and relative frequency (percentage, %); 0—No, 1—Yes.

**Table 3:** Resistance patterns of AOP pathogens.

Aspect	*E. coli*		*Enterococcus* spp.		*Klebsiella* spp.		*Proteus* spp.		*Pseudomonas* spp.		Wilks’ G^2^ Test *	
	*n*	%	*n*	%	*n*	%	*n*	%	*n*	%	*p*-Value	V
	58		7		21		9		5			
ESBL Producer												
ESBL (−)	43	74.14	7	100.00	18	85.71	9	100.00	5	100.00	0.030	0.269
ESBL (+)	15	25.86	0	0.00	3	14.29	0	0.00	0	0.00		
MDR status												
MDR No	39	67.24	5	71.43	14	66.67	9	100.00	4	80.00	0.138	0.211
MDR Yes	19	32.76	2	28.57	7	33.33	0	0.00	1	20.00		
Classes of antibiotics												
Penicillins	0	0.00	5	71.43	0	0.00	0	0.00	0	0.00	<0.001	0.702
Carbapenems	15	25.86	0	0.00	3	14.29	0	0.00	0	0.00		
Cephalosporins	0	0.00	0	0.00	14	66.67	0	0.00	5	100.00		
Combination	0	0.00	0	0.00	4	19.05	0	0.00	0	0.00		
Fluoroquinolones	43	74.14	0	0.00	0	0.00	9	100.00	0	0.00		
Glycopeptides	0	0.00	2	28.57	0	0.00	0	0.00	0	0.00		

ESBL—Extended-spectrum beta-lactamase; MDR—multidrug resistance; * alternative of Chi-square test; V = Cramer’s V Coefficient—a measure of association between two nominal variables. Its value ranges from 0 to 1. Cramer’s V indicates the strength of the association between two categorical variables. V < 0.1 (negligible), V = 0.1–0.3 (low/moderate), V > 0.3 (moderate/strong).

**Table 4:** Associations between antibiotic drugs and bacterial pathogens.

	Pathogen	*E. coli*		*Enterococcus* spp.		*Klebsiella* spp.		*Proteus* spp.		*Pseudomonas* spp.	
Antibiotic		r*ij	*p*-Value	r*ij	*p*-Value	r*ij	*p*-Value	r*ij	*p*-Value	r*ij	*p*-Value
Ampicillin		−2.696	0.011	**8.362**	<0.0001	−1.183	0.58	−0.721	1.000	−0.526	1.000
Cefepime		−2.696	0.011	−0.629	1.000	−1.183	0.581	−0.721	1.000	**10.000**	<0.0001
Ceftriaxone		−4.741	<0.0001	−1.107	0.589	**7.826**	<0.0001	−1.269	0.352	−0.926	1.000
Ciprofloxacin		**5.207**	<0.0001	−2.856	0.005	−5.366	<0.0001	**3.021**	0.003	−2.388	0.023
Ertapenem		−2.067	0.071	−0.482	1.000	**3.411**	0.008	−0.553	1.000	−0.403	1.000
Meropenem		**3.575**	0.000	−1.153	0.590	−2.166	0.036	−1.321	0.348	−0.964	1.000
Piperacillin- tazobactam		−2.399	0.029	−0.560	1.000	**3.959**	0.002	−0.642	1.000	−0.468	1.000
Vancomycin		−1.679	0.174	**5.207**	0.004	−0.737	1.000	−0.449	1.000	−0.328	1.000

r*ij = adjusted Pearson residuals; in a significant association, r*ij value is usually >1.96; Statistical tool: Chi-square test, adjusted Pearson residuals, and Fisher’s exact test. Bolded values are statistically significant at *p* < 0.05.

**Table 5:** ESBL status of AOP bacteria identified in renal pelvis urine cultures, drainage type, and clinical outcomes.

Aspect	Total	ESBL (+)		ESBL (-)		Chi-Square Test	
	*n* = %	*n*	%	*n*	%	*p*-Value	OR/V
	100	18		82			
MDR Status							
MDR No	71	0	0	71	86.58	<0.0001	V = 1
MDR Yes	29	18	100	11	13.41		
Drainage type							
Nephrostomy	56	13	72.22	43	52.44	0.966	OR = 1.272
Ureteral stent	44	5	27.78	39	47.56		
ICU Admission							
ICU No	78	16	88.89	62	75.61	0.773	OR = 1.016
ICU Yes	22	2	11.11	20	24.39		
iHM status							
iHM No	99	17	100.00	82	98.78	0.032	V = 0.733
iHM Yes	1	1	0.00	0	1.22		

*n*—frequency, %—relative frequency; *p* < 0.05—statistically significant differences; ESBL—extended spectrum beta-lactamase; ICU—intensive care unit; iHM—intrahospital mortality; OR—odd ratio, a statistical measure used to quantify the strength and direction of the association between two categorical variables: positive correlation (OD > 1), negative correlation (OD < 1), no correlation (OD = 1); V = Cramer’s V Coefficient, that indicates the strength of the association between two categorical variables. Its value ranges from 0 to 1: V < 0.1 (negligible), V = 0.1–0.3 (low/moderate), V > 0.3 (moderate/strong).

**Table 6:** Laboratory analyses and clinical evolution of patients with AOP.

	*E. coli*	*Enterococcus* spp.	*Klebsiella* spp.	*Proteus* spp.	*Pseudomonas* spp.
WBC/mm^3^					
Mean	14,615.02	14,211.43	14,998.29	15,730.67	16,406.00
SD	2863.21	1160.74	2763.26	3952.63	2896.18
CRP (mg/mL)					
Mean	194.21	186.84	202.80	182.79	231.10
SD	67.31	70.14	50.85	66.39	65.73
PCT (ng/mL)					
Median	5.00	6.60	3.70	3.90	3.50
IQR	3.13–8.15	4.80–8.70	2.50–4.70	3.40–6.30	2.20–4.50
ESR (mm/h)					
Mean	62.00	68.57	62.76	60.89	67.00
SD	16.88	14.21	21.40	13.92	8.00
Fibrinogen (mg/dL)					
Mean	453.76	515.57	458.67	463.56	442.00
SD	73.52	92.61	74.39	97.74	65.43
LMR					
Mean	3.33	3.04	2.92	2.97	3.19
SD	1.13	1.08	1.03	0.94	1.04
Time to fever resolution (hours)					
Mean	64.82	60.29	60.70	55.24	59.82
SD	17.46	15.14	19.14	7.84	10.82
Clinical improvement (days)					
Mean	3.21	2.43	3.12	3.26	2.58
SD	1.22	1.10	1.15	1.03	0.72
IV Switch to Oral (days)					
Median	4.00	3.60	3.60	3.80	3.30
IQR	3.00–5.30	3.10–4.10	3.00–4.60	3.10–4.10	3.30–5.80
Hospitalization period (days)					
Mean	7.62	7.86	7.52	8.22	9.00
SD	2.25	3.04	2.20	1.69	1.79
Antibioterapy duration (days)					
Mean	10.91	11.29	10.81	9.56	11.20
SD	3.37	3.10	2.86	1.89	2.93

WBC—white blood cells; CRP—C-reactive protein; PCT—procalcitonin; ESR—erythrocyte sedimentation rate; LMR—lymphocyte-to-monocyte ratio; SD—standard deviation; IQR—interquartile range.

**Table 7:** The correlations between the clinical evolution of patients with AOP and baseline data, symptoms, imaging features, obstruction cause, and pathogen type.

	Residence	Recurrent Lithiasis	Fever	Obstruction Cause	HDN	Bilateral Involvement	Pyonephrosis	ESBL	MDR
Time to fever resolution (hours)									
*p*-value	0.983	0.498	**0.028**	**0.006**	0.079	0.165	**0.028**	**<0.0001**	**0.022**
Clinical improvement (days)									
*p*-value	0.282	0.827	0.662	0.641	0.270	0.546	0.283	**0.003**	0.896
IV Switch to Oral (days)									
*p*-value	0.077	**0.027**	0.813	**0.044**	**0.009**	**0.035**	0.665	**<0.0001**	**0.019**
Antibiotic duration (days)									
*p*-value	**0.009**	0.615	0.998	**0.035**	0.095	0.848	0.228	0.351	0.928

HDN—Hydronephrosis; ESBL—Extended-spectrum beta-lactamase; MDR—multidrug resistance; IV—intravenous. Bolded values are statistically significant, *p* < 0.05. Statistical tool: MANOVA.

## Data Availability

Due to the ongoing study, the data presented in this study are available upon request from the first author and the corresponding author.

## Abbreviations

The following abbreviations are used in this manuscript:

AOP	Acute obstructive pyelonephritis
ESBL	Extended-spectrum beta-lactamases
MDR	Multidrug-resistant
WBC	White blood cells
CRP	C-reactive protein
CKD	Chronic kidney disease
T2DM	Type 2 diabetes mellitus
HDN	Hydronephrosis
PCT	Procalcitonin
ESR	Erythrocyte sedimentation rate
LMR	Lymphocyte-to-monocyte ratio
PBUC	Bladder urine culture
RPUC	Renal pelvis urine culture
SD	Standard deviation
OD	Odds Ratio
V	Cramer’s V coefficient
r*ij	Adjusted Pearson residuals
CFU	Colony-forming units
ICU	Intensive care unit
ECU	Emergency care unit
iHM	Intrahospital mortality
